# EUS is accurate in characterizing pancreatic cystic lesions; a prospective comparison with cross-sectional imaging in resected cases

**DOI:** 10.1007/s00464-020-08166-3

**Published:** 2020-12-01

**Authors:** Sahar Wesali, Mehmet A. Demir, Caroline S. Verbeke, Mats Andersson, Svein Olav Bratlie, Riadh Sadik

**Affiliations:** 1 Department of Gastroenterology and Hepatology, Sahlgrenska University Hospital, Gothenburg, Sweden; 2 Department of Clinical Pathology, Rigshospitalet, Copenhagen, Denmark; 3 Department of Pathology, University of Oslo, and Oslo University Hospital, Oslo, Norway; 4 Department of Radiology, Sahlgrenska University Hospital, Gothenburg, Sweden; 5 Division of Radiology, Karolinska University Hospital Huddinge, Stockholm, Sweden; 6 Department of Surgery, Sahlgrenska University Hospital, Gothenburg, Sweden

**Keywords:** Pancreatic cystic lesions, IPMN, MCN, Pancreatic adenocarcinoma, EUS-FNA, Cross-sectional imaging

## Abstract

**Background:**

Imaging modalities for characterizing pancreatic cystic lesions (PCLs) is a known uncertainty. The aim of this prospective study was to compare the diagnostic performance of endoscopic ultrasound morphology, cytology and cyst fluid carcinoembryonic antigen (EUS-FNA-CEA) with cross-sectional imaging in resected PCLs.

**Methods:**

The cross-sectional imaging and EUS-FNA-CEA results were collected in an academic tertiary referral centre using histology of the surgical specimen as the diagnostic standard.

**Results:**

Of 289 patients undergoing evaluation for PCL with cross-sectional imaging and EUS-FNA between February 2007 and March 2017, 58 underwent surgical resection providing a final diagnosis of the PCLs: 45 mucinous, 5 serous, 1 pseudocyst, 2 endocrine, 2 solid pseudopapillary neoplasms and 3 other. EUS-FNA-CEA was more accurate than cross-sectional imaging in diagnosing mucinous PCLs (95% vs. 83%, *p* = 0.04). Ninety-two percent of the PCLs with high-grade dysplasia or adenocarcinoma were smaller than 3 cm in diameter. The sensitivity of EUS-FNA-CEA and cross-sectional imaging for detecting PCLs with high-grade dysplasia or adenocarcinoma were 33% and 5% (*p* = 0.03), respectively. However, there was no difference in accuracy between the modalities (62% vs. 66%, *p* = 0.79). The sensitivity for detecting pancreatic adenocarcinomas only was 64% for EUS-FNA-CEA and 9% for cross-sectional imaging (*p* = 0.03). Overall, EUS-FNA-CEA provided a correct diagnosis in more patients with PCLs than cross-sectional imaging (72% vs. 50%, *p* = 0.01).

**Conclusions:**

EUS-FNA-CEA is accurate and should be considered a complementary test in the diagnosis of PCLs. However, the detection of PCLs with high-grade dysplasia or adenocarcinoma needs to be improved. Cyst size does not seem to be a reliable predictor of high-grade dysplasia or adenocarcinoma.

**Electronic supplementary material:**

The online version of this article (10.1007/s00464-020-08166-3) contains supplementary material, which is available to authorized users.

With the increased use of advanced cross-sectional imaging techniques in recent decades, incidentally discovered cysts in the pancreas have become more common [1]. Pancreatic cystic lesions (PCLs) constitute a heterogeneous group of tumours that can be benign, premalignant or malignant [2]. The prevalence of PCLs has been estimated to range from 2.6% to 19.6% in cross-sectional imaging studies [3–5]. Modern imaging techniques are capable of detecting these lesions but may often not be able to distinguish malignant from benign lesions [6]. The prognosis of pancreatic cancer is poor, and only 20% are eligible for potentially curative surgery [7]. Therefore, early detection and preventive pancreatic surgery are key to improving outcomes. At the same time, the small risk of malignant transformation, the high risks associated with surgical treatment, the limitations of diagnostic modalities and the lack of high-quality prospective studies have led to contradictory recommendations for the management of PCLs [8].

Multidetector row computed tomography (CT) offers thin section technique that can provide detailed information on a cyst’s structure and is considered an initial method of good quality for the characterization of PCLs [9]. The advantage of magnetic resonance imaging (MRI) is its superior contrast resolution that facilitates the recognition of duct communication with the cyst [10]. However, previous data suggest that cross-sectional imaging with CT and MRI performs comparably regarding the characterization of PCLs [11]. According to earlier studies, the accuracy of CT and MRI in diagnosing PCLs correctly is 40–60% [5, 11].

Endoscopic ultrasound (EUS) provides high-resolution imaging of PCLs [12]. In addition, EUS allows fine-needle aspiration (FNA) for analyses based on cytology, biochemistry and tumour markers of cyst fluid. EUS-guided FNA is reported to provide a correct diagnosis in 62–97% of cases [13–15].

While there are several published studies comparing the diagnostic value of EUS and cross-sectional imaging in patients with PCLs [16, 17], few prospective trials have compared the two imaging modalities [18, 19]. These studies are either based on small study populations or compare assessments of detailed structures of PCLs without taking into consideration the added findings of cyst fluid analysis. The overall purpose of this prospective study was to compare the diagnostic performance of EUS-FNA with cyst fluid analysis and that of cross-sectional imaging (CT/MRI) in surgically resected PCLs. The main focus was the diagnostic accuracy of the two modalities in detecting mucinous PCLs and high-grade dysplasia or adenocarcinoma in PCLs.

## Methods and materials

### Patients

All patients with suspected PCLs that were identified with cross-sectional imaging and referred for EUS-FNA at Sahlgrenska University Hospital between February 2007 and March 2017 were consecutively enrolled. The catchment area of this tertiary referral centre in Western Sweden includes two million inhabitants. The only inclusion criterion was the presence of a PCL on EUS. Patients without a definite histology diagnosis based on a resection specimen were excluded.

The study was approved by the Regional Ethics Committee in Gothenburg, Sweden, with the registration number 555-07. Patients gave written consent to participate in the study after oral and written information was provided to them. The trial was registered in the ClinicalTrials.gov database (NCT03884179) and was conducted according to the Standards for Reporting of Diagnostic Accuracy Studies (STARD 2015) guidelines.

### Cross-sectional imaging

All PCLs were initially diagnosed by CT or MRI at different hospitals in the catchment area. The images were subsequently re-examined at a multidisciplinary therapy (MDT) conference at Sahlgrenska University Hospital by radiologists with pancreatobiliary expertise. The radiological assessment at Sahlgrenska was made according to the International consensus guidelines for the management of intraductal papillary mucinous neoplasms (IPMNs) and mucinous cystic neoplasms (MCNs) of the pancreas [20–22].

### EUS methods

A linear echoendoscope (EG3870UTK, Pentax, Tokyo, Japan) was used to perform the EUS examination under conscious sedation. The PCL was accessed by the transgastric/transduodenal route using a 22/25-gauge needle (Wilson-Cook/Olympus/Boston Scientific). The cyst fluid was first aspirated for the analysis of CEA. Then, the needle was moved very gently within the cyst for 60–120 s under aspiration. A cytopathology technician was present and created a smear on a piece of glass, and the rest of the yield was sent in ThinPrep fluid to the cytopathologist. If there was enough cyst fluid, the amylase level was analysed as well.

### EUS morphology

All EUS examinations were performed by the same endoscopist, who diagnosed the PCLs according to the presence or absence of the following morphologic findings: (1) macrocystic/microcystic septations, (2) solid components, (3) a thick wall, (4) suspected mucin, (5) communication with a pancreatic duct, (6) dilatation of the main pancreatic duct, (7) a mucus plug in the papilla and (8) hypervascularity. The EUS features were previously defined by Gress et al. [23].

### Cytology

Cytology with periodic acid-Schiff staining for mucus was performed and evaluated by dedicated cytopathologists at Sahlgrenska University Hospital. Diagnostic samples were classified based on the presence or absence of (1) mucin, (2) representative cell groups from the lesion and (3) dysplasia.

### CEA

The cyst fluid concentration of CEA was analysed using immunochemiluminescence. As shown before, a CEA cut-off of > 192 ng/ml was applied for mucinous PCLs [14, 24], and that of > 1000 ng/ml was applied for the assessment of high-grade dysplasia or pancreatic adenocarcinoma [25]. A CEA value of 5 ng/ml or less was considered indicative of a serous PCL [26].

### Surgery

**Fig. 1:**
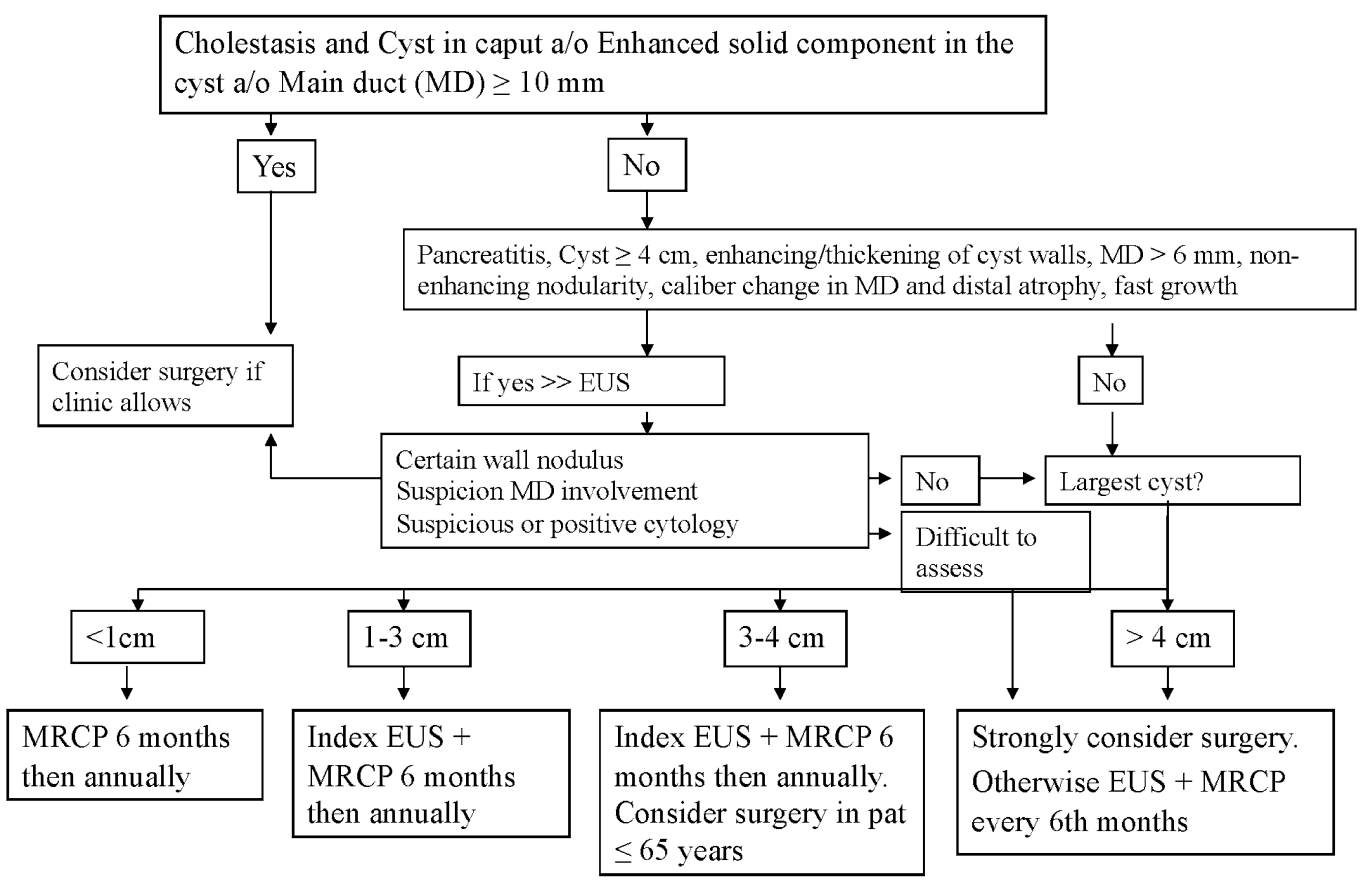
Algorithm for the management of branch duct intraductal papillary mucinous neoplasms (BD-IPMNs). *MRCP* magnetic resonance cholangiopancreatography, *EUS* endoscopic ultrasound

After cross-sectional imaging and EUS-FNA were performed, the assessment of the PCL was made by the members of the MDT team at Sahlgrenska University Hospital, which included surgeons, radiologists and oncologists. The decision to operate was made according to the local guidelines on the management of branch duct IPMN (BD-IPMN) (Fig. 1), which were adapted from *Tanaka *et al.[20, 21] and the European experts consensus statement [27]. Other indications for surgery included main duct IPMN (MD-IPMN), MCN, adenocarcinoma, neuroendocrine tumour (NET) and solid pseudopapillary neoplasm (SPN). Even large and symptomatic benign cystic lesions were considered for surgery.

### Histology

Dedicated pathologists at the Sahlgrenska University Hospital and Oslo University Hospital evaluated the surgical specimens. The diagnoses of the resected PCLs were based on the final pathology reports.

### Definitions and outcome

Cross-sectional imaging refers to the results of CT or MRI for the diagnosis of PCLs. The term EUS-FNA-CEA was used when a combination of EUS morphology, cytology and/or CEA was used to make a diagnosis. A PCL was regarded as mucinous if any of the above-mentioned EUS test results were positive. The only exception was the presence of a CEA value of 5 ng/ml or less, which is highly indicative of a serous cystic lesion [26]. Similarly, if any of these three EUS modalities indicated high-grade dysplasia or adenocarcinoma, the PCL was regarded as such by EUS-FNA-CEA. The final pathology report of the resection specimen was considered the gold standard against which cross-sectional imaging and EUS-FNA-CEA were compared.

*The first outcome* measure was the diagnostic performance of EUS-FNA-CEA for mucinous PCLs, which was compared with the three EUS tests alone (EUS morphology, cytology and CEA). *The second outcome* was the diagnostic accuracy measures of cross-sectional imaging for mucinous PCLs, which was then compared with that of EUS-FNA-CEA. *The third outcome* measures were the diagnostic performance of EUS-FNA-CEA and cross-sectional imaging for the detection of high-grade dysplasia or pancreatic adenocarcinoma. These were compared with each other. *The fourth outcome* measures were the same as the third but for pancreatic adenocarcinoma only. *The fifth outcome* measure was the resulting adverse events.

### Statistical methods

Statistical analyses were performed using SPSS for Mac version 24.0. Descriptive statistics, including means, medians and ranges, where appropriate, were calculated for all variables. Nonparametric statistics were applied with McNemar’s test to compare the differences between methods within the same patient group. A *p* value of < 0.05 was considered statistically significant, and the *p* values were adjusted by the Bonferroni method when appropriate. When tests of statistical significance were applied to compare the sensitivity, specificity and accuracy rates, patients with indeterminate test results were grouped with the negative results [28]. Considering the paired design, the estimated sample size was determined to be 52 using the methodology published by Alonzo et al. [29]. This sample size would allow 80% power to detect differences in sensitivity and specificity between cross-sectional imaging and EUS-FNA at the 5% (two-sided) significance level. The expected effect size was estimated from the results of the previous studies.

## Results

**Fig. 2:**
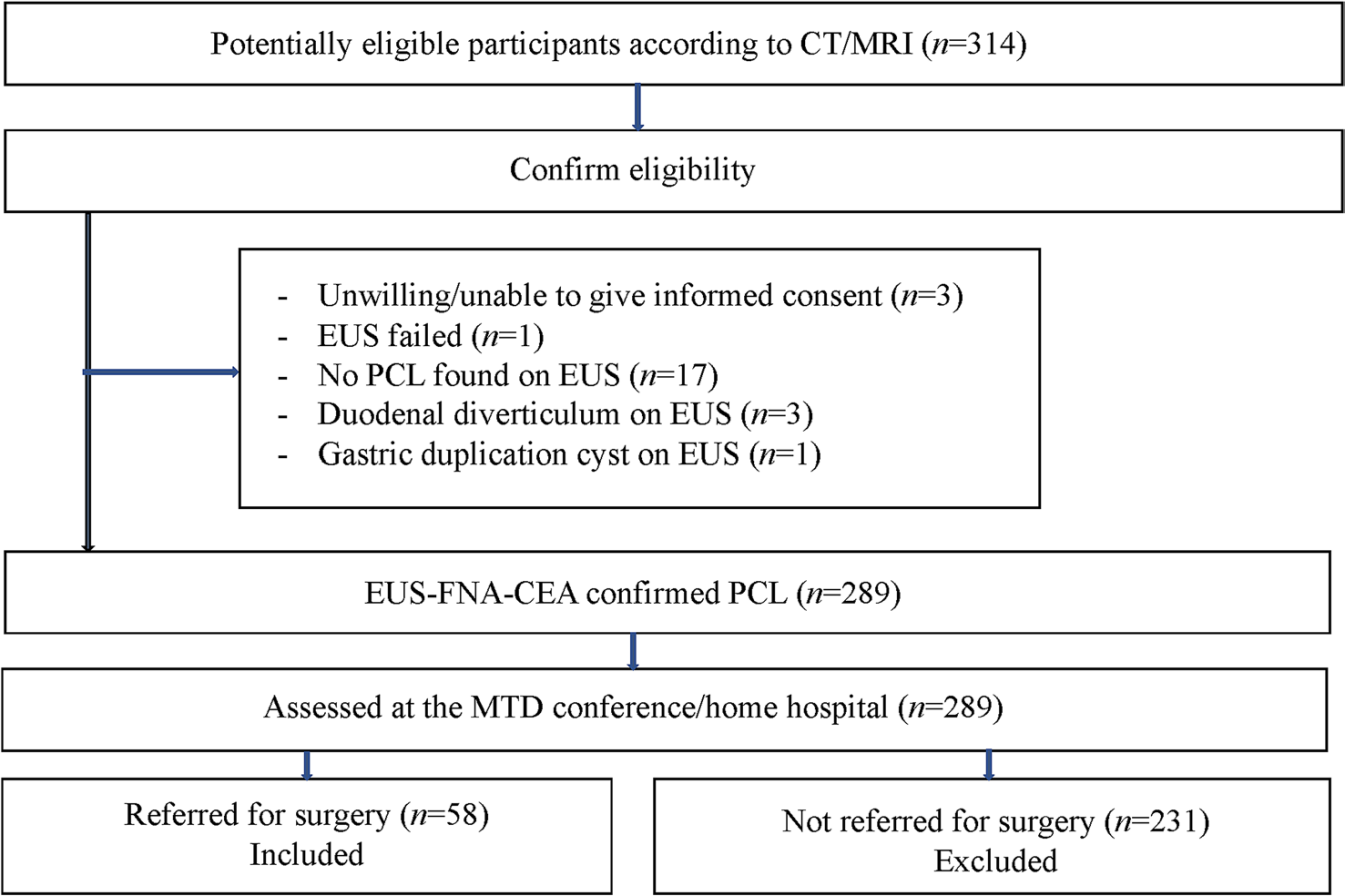
Flow diagram of patient inclusion/exclusion. *CT* computed tomography, *MRI* magnetic resonance imaging, *EUS-FNA-CEA* endoscopic ultrasound morphology, cytology and carcinoembryonic antigen, *MDT* multidisciplinary therapy

Between February 2007 and March 2017, 289 patients with PCLs identified with cross-sectional imaging underwent EUS-FNA, which confirmed the presence of a cystic lesion. The mean duration between the cross-sectional imaging and EUS tests was 2.5 months (range, 0–10 months). The inclusion/exclusion process of patients is shown in the flow chart in Fig. 2.

Fifty-eight patients underwent pancreatic resection, yielding definitive histologic diagnoses of the PCLs. The patient characteristics are presented in Table 1. In brief, the median age was 68.0 years, and the majority of patients were female (62%). The PCLs were predominately mucinous (78%). The patients with mucinous and non-mucinous PCLs had a mean age of 66.9 and 56.9 years, respectively. They had the same sex distribution (62% female patients). The characteristics of the PCLs according to EUS are presented in Table 2. Mucinous PCLs were more often located in the pancreatic head (74%), and they were often smaller than 3 cm (77%). Ninety-two percent of the PCLs with high-grade dysplasia or adenocarcinoma were smaller than 3 cm in diameter.

**Table 1:** Baseline characteristics of the included patients with PCLs (*n* = 58)

Pathological diagnosis (*n*)	Subjects (*n*)	Age (years, mean)	Sex (*n*)		Examinations performed (*n*)		
			Female	Male	CT	MRI	EUS
Mucinous (LGD/IGD**)**	24	67.3	13	11	1	23	24
Mucinous (HGD/ Adenocarcinoma)	21	66.4	15	6	6	15	21
Serous	5	58.4	3	2	2	3	5
Pseudocyst	1	53.0	1	0	1	0	1
Endocrine	2	62.0	0	2	0	2	2
Solid pseudopapillary	2	36.5	2	0	0	2	2
Other	3	66.0	2	1	1	2	3
Total	58	64.7	36	22	11	47	58

*CT* computed tomography, *MRI* magnetic resonance imaging, *EUS* endoscopic ultrasound; *LGD* low-grade dysplasia, *IGD* intermediate-grade dysplasia, *HGD* high-grade dysplasia

**Table 2:** Characteristics of the PCLs according to EUS

Pathological diagnosis (*n*)	Location of cyst (*n*)					Size of cyst (mm)			
	Head	Body	Tail	Multifocal	Not reported^a^	< 15	15–30	> 30	Not reported^a^
Mucinous (LGD/IGD)	12	2	3	1	6	1	10	6	7
Mucinous (HGD/ Adenocarcinoma)	13	1	2	0	5	4	8	1	8
Serous	2	0	1	2	0	0	0	5	0
Pseudocyst	0	0	0	1	0	0	0	1	0
Endocrine	0	1	1	0	0	0	0	2	0
Solid pseudopapillary	1	0	0	1	0	0	1	1	0
Other	0	1	1	1	0	1	1	1	0
Total	28	5	8	6	11	6	20	17	15

*LGD* low-grade dysplasia, *IGD* intermediate-grade dysplasia, *HGD* high-grade dysplasia, *size of cyst* maximum cyst diameter

^a^If the main pancreatic duct was targeted, the cyst size or location was not reported

Appendix Table 1 in the Supplementary Material provides an overview of all patients included in the study.

### Diagnosing mucinous PCLs

**Fig. 3:**
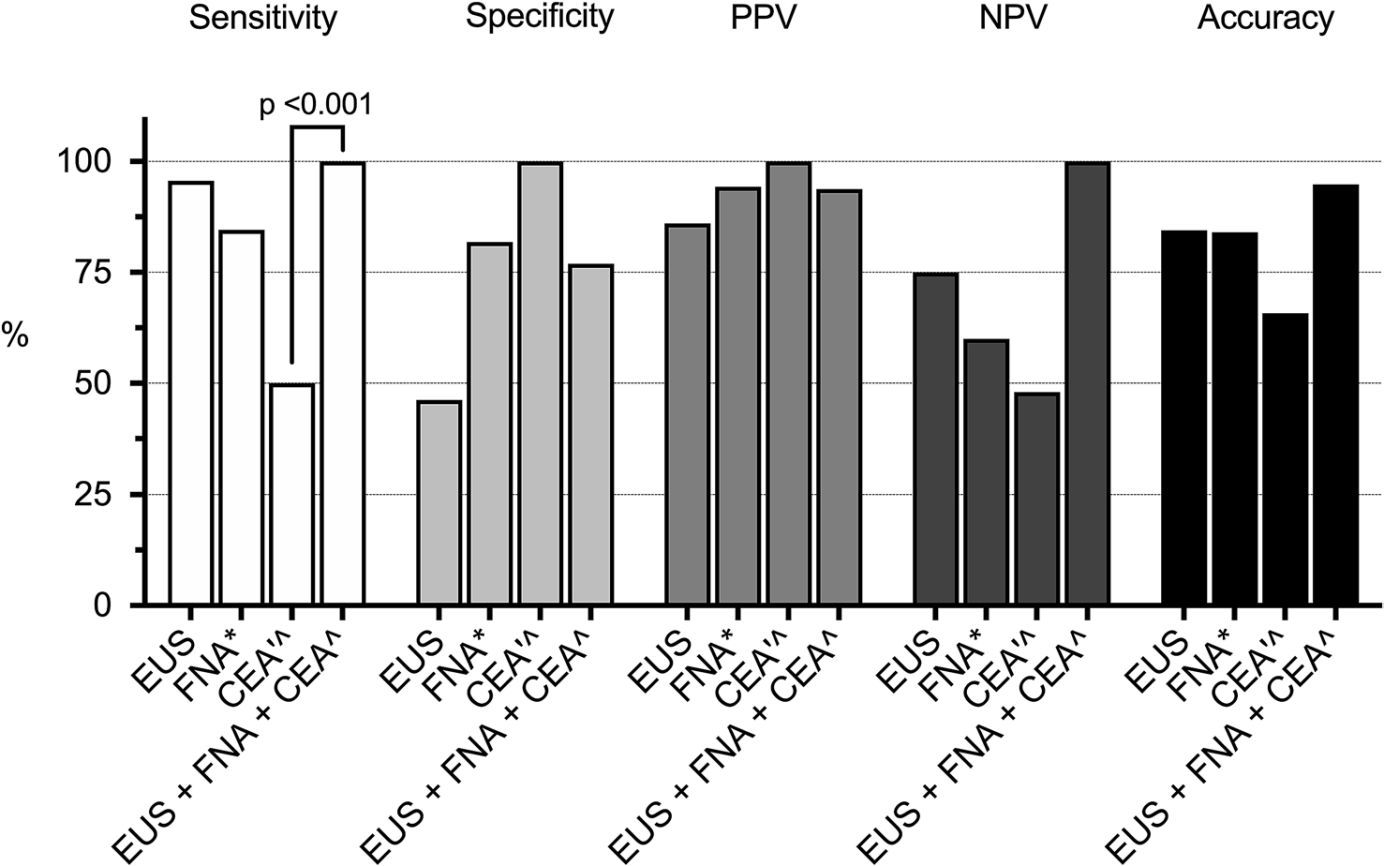
Performance of EUS morphology, cytology and CEA in the diagnosis of mucinous PCLs. *Eight patients did not have a diagnostic cytology report. ´Nineteen patients did not have a CEA result. ^Cut-off value of > 192 ng/ml indicates mucinous PCLs. *EUS-FNA-CEA* endoscopic ultrasound morphology, cytology and carcinoembryonic antigen, *PPV* positive predictive value, *NPV* negative predictive value, *Mucinous PCLs* main/branch duct intraductal papillary mucinous neoplasm (M/BD-IPMN), mucinous cystic neoplasm (MCN) or adenocarcinoma

The performance of EUS in the diagnosis of mucinous PCLs is illustrated in Fig. 3. EUS morphology provided a diagnosis in all patients but cytology and CEA were diagnostic in 86% and 67% of cases, respectively. EUS morphology showed a sensitivity of 96% for diagnosing mucinous lesions. The presence of a mucus plug in the duodenal papilla was 100% (5 out of 5), indicative of an MD-IPMN. The cytology and CEA results showed a higher specificity (82% and 100%, respectively) in the differentiation of mucinous and non-mucinous PCLs than did EUS morphology. However, the differences did not reach statistical significance. EUS-FNA-CEA showed a significantly higher sensitivity (100%) for diagnosing mucinous PCLs compared with CEA. The overall accuracy of EUS-FNA-CEA in the diagnosis of mucinous PCLs was not significantly higher than those of EUS morphology, cytology or CEA alone when the results were adjusted with the Bonferroni correction for multiple comparisons (*p* = 0.09, *p* = 0.13 and *p* = 0.06, respectively).

The CEA concentrations in mucinous and non-mucinous PCLs are presented in the Appendix Fig. 1 in the Supplementary Material. Since the sensitivity of cyst CEA in detecting mucinous PCLs at a cut-off value of > 192 ng/ml was low, a ROC curve analysis was performed to determine the optimal cut-off value for CEA for differentiating between mucinous and non-mucinous PCLs. This is presented in the Appendix Fig. 2 in the Supplementary Material. A CEA value of > 11.5 ng/ml provided the best sensitivity (0.96) and a moderate specificity (0.75) (AUC 0.899). Then a binary logistic regression was performed, which showed that those with CEA levels higher than 11.5 ng/ml had a significantly higher risk of having mucinous PCLs (odds ratio 75; 95% CI 6.9–816.9, *p* < 0.001).

Cyst fluid amylase levels were possible to analyse in 38% (22 out of 58) of patients and are presented in the Appendix Table 2 in the Supplementary Material.

**Fig. 4:**
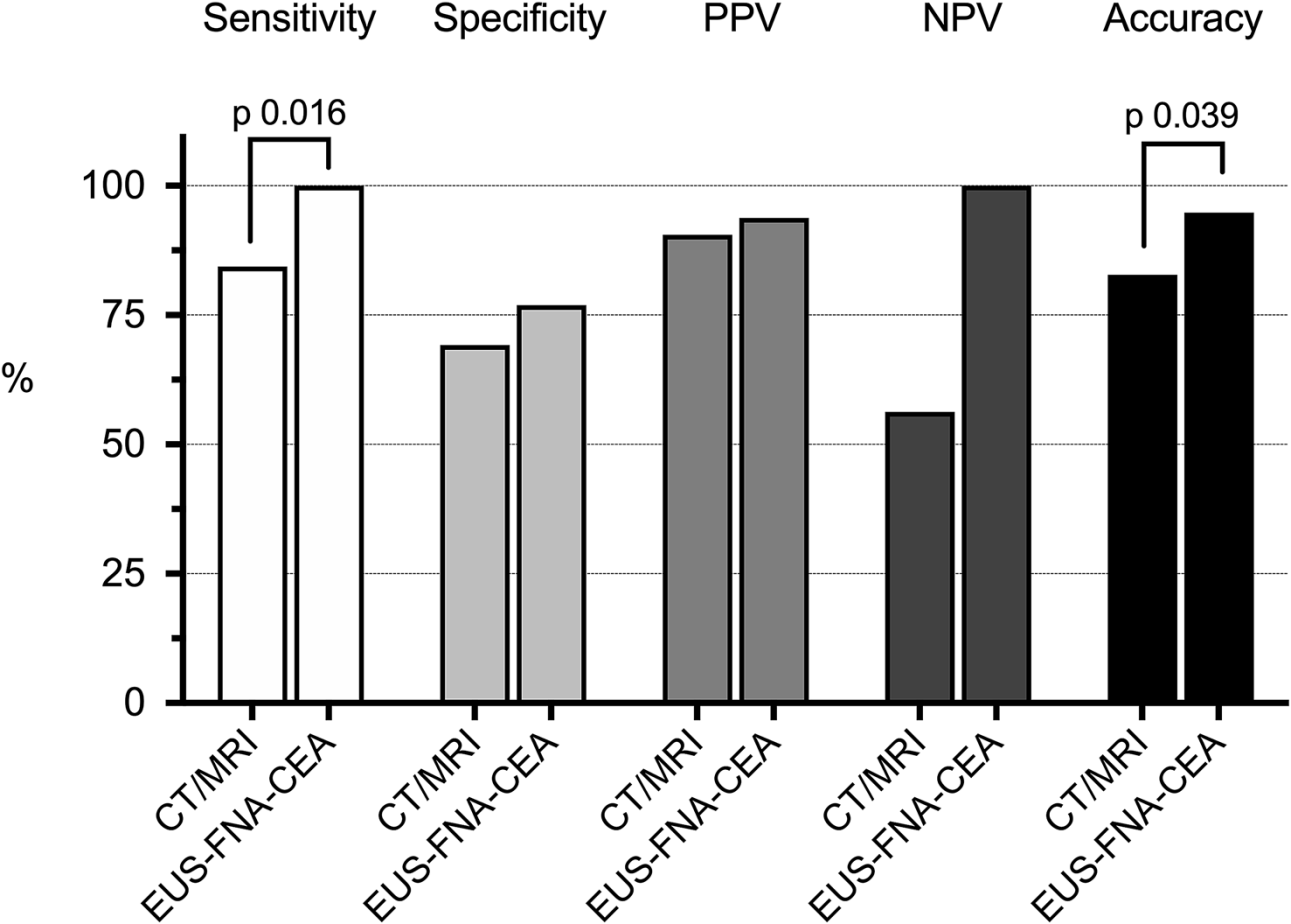
Performance of cross-sectional imaging and EUS-FNA-CEA in the diagnosis of mucinous PCLs. *CT* computed tomography, *MRI* magnetic resonance imaging, *EUS-FNA-CEA* endoscopic ultrasound morphology, cytology and carcinoembryonic antigen, *PPV* positive predictive value, *NPV* negative predictive value, *Mucinous PCLs* main/branch duct intraductal papillary mucinous neoplasm (M/BD-IPMN), mucinous cystic neoplasm (MCN) or pancreatic adenocarcinoma

Cross-sectional imaging had a sensitivity rate of 38 of 45 (84%) for diagnosing mucinous PCLs, with a specificity of 9 of 13 (69%) (Table 3). EUS-FNA-CEA had a significantly higher sensitivity and overall accuracy for detecting mucinous PCLs compared with cross-sectional imaging (Fig. 4).

**Table 3:** Performance of cross-sectional imaging in the diagnosis of mucinous PCLs

	Pathological diagnosis (*n*)	
	Mucinous	Non-mucinous
CT/MRI result*s* (*n*)		
Mucinous	38	4
Inconclusive	7	5
Non-mucinous	0	4

*CT* computed tomography, *MRI* magnetic resonance imaging

### Diagnosing PCLs with high-grade dysplasia or adenocarcinoma

**Fig. 5:**
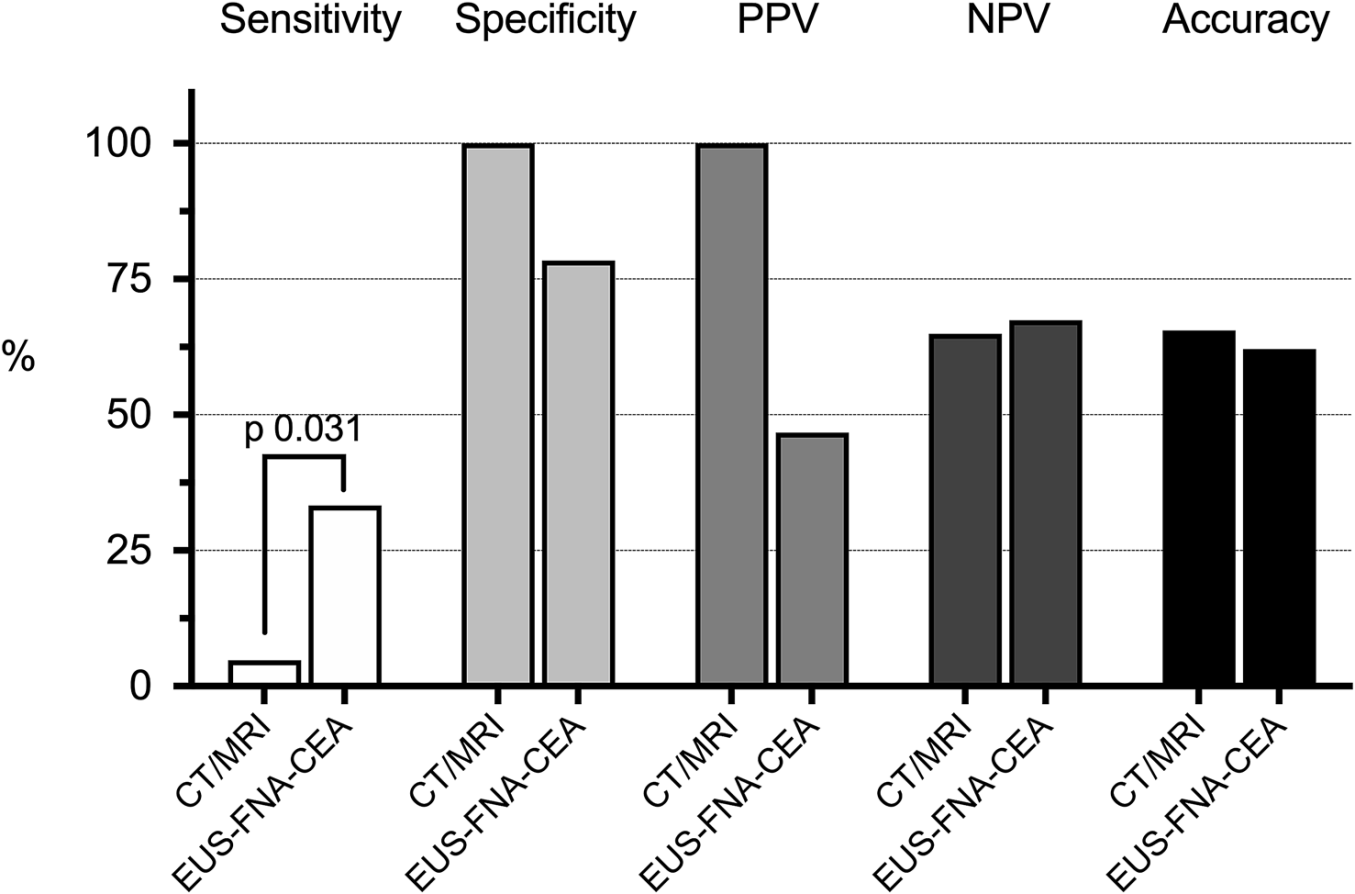
Performance of cross-sectional imaging and EUS-FNA-CEA in the diagnosis of HGD/pancreatic adenocarcinoma. *CT* computed tomography, *MRI* magnetic resonance imaging, *EUS-FNA-CEA* endoscopic ultrasound morphology, cytology and carcinoembryonic antigen, *PPV* positive predictive value, *NPV* negative predictive value, *HGD* high-grade dysplasia

The sensitivity of cross-sectional imaging in the diagnosis of PCLs with high-grade dysplasia or adenocarcinoma was significantly lower than that of EUS-FNA-CEA (5% vs. 33%, *p* = 0.03) (Fig. 5). However, there was no difference in the overall accuracy between the modalities (66% vs. 62%, *p* = 0.79). There were 11 pancreatic adenocarcinomas found in the study. The sensitivity for detecting pancreatic adenocarcinomas was 64% for EUS-FNA-CEA and 9% for cross-sectional imaging (*p* = 0.03). Further analysis did not show any difference in accuracy between EUS-FNA-CEA and cross-sectional imaging for diagnosing adenocarcinomas (67% vs. 83%, *p* = 0.21).

### Diagnosing the entity of a PCL

The EUS-FNA-CEA results provided a conclusive diagnosis for all PCLs, but cross-sectional imaging yielded inconclusive diagnoses in 12 patients (Table 3). This result created a type two error, as inconclusive test results were grouped with the negative results. EUS-FNA-CEA yielded a correct diagnosis in 67% (8 out of 12) of the patients with PCLs that had inconclusive cross-sectional imaging results. Overall, EUS-FNA-CEA provided a correct diagnosis in more patients with PCLs than did cross-sectional imaging (72% vs. 50%, *p* = 0.01). 

### Adverse events

No adverse events as a result of cross-sectional imaging were reported in the patients included in this study. Two patients developed transient vasovagal reaction during EUS-FNA, which was successfully treated with atropine and fluids intravenously. One patient complained of abdominal pain within 12 h after EUS-FNA. The physical examination results were normal, and the blood tests showed only a slight elevation in pancreatic amylase of less than three times above the reference range. No patients required inpatient care.

## Discussion

This 10-year-long study is unique because it prospectively compared the diagnostic performance of cross-sectional imaging and EUS-FNA with cyst fluid analysis for PCLs. Consequently, it has illustrated the overall role of both modalities in the assessment of PCLs. The study has also demonstrated the value of combining EUS morphology, cytology and CEA to improve diagnostic accuracy. To provide the best gold standard for the assessment of diagnostic accuracy, only cases with resected lesions were included.

The demographics of the patients with PCLs who underwent surgical resection in this study are comparable with those reported in the previous studies [14, 30]. An unexpected result is that PCLs with high-grade dysplasia or adenocarcinoma were likely to be smaller than 3 cm. This new knowledge should influence any guidelines developed in the future, as the present recommendations are based mainly on the size of the cysts.

EUS morphology alone showed a very high sensitivity for identifying mucinous PCLs, which is inconsistent with the results of some previous studies [13, 14]. An explanation for this result may be that the same experienced endoscopist performed all EUS examinations. The sensitivity of CEA in detecting mucinous PCLs, at a cut-off value of > 192 ng/ml, was low, which is contradictory to earlier findings [14]. The inconsistency in the results may be due to different CEA assays being used [31]. Conversely, the specificity of CEA at this level was very high. The distribution of data in the boxplot in the Appendix Fig. 1 indicates that a cut-off value of less than 192 ng/ml might be superior for distinguishing between mucinous and non-mucinous PCLs. The ROC curve analysis performed suggests that a lower cut-off value of > 11.5 is optimal for differentiating between mucinous and non-mucinous PCLs. Additional studies based on larger study populations are needed to identify the optimal cut-off value for CEA in the diagnosis of PCLs.

The differences in accuracy between EUS-FNA-CEA and any of the EUS tests alone in the diagnosis of mucinous PCLs approximated statistical significance. These results support the use of the combination of EUS morphology, cytology and CEA for diagnosing PCLs. This strategy has previously been reported by Frossard et al. [13]. In clinical practice, it is not always possible to obtain sufficient fluid for CEA or cytology tests. In this study, only 39 of 58 patients with PCLs had a CEA result. This result also indicates the need to aim for the combination of EUS morphology, cytology and CEA, rather than a single test, in order to ensure adequate results in the diagnostic work-up of PCLs.

The overall accuracy of cross-sectional imaging in the diagnosis of mucinous PCLs (83%) was better in this study than that previously reported [5, 11]. However, EUS-FNA-CEA was more accurate in the distinction between mucinous and non-mucinous PCLs. Unlike cross-sectional imaging, EUS-FNA-CEA was conclusive in all examinations, and overall, it had a higher diagnostic yield. This result indicates the benefit of performing EUS-FNA-CEA as a complementary test to cross-sectional imaging to improve the diagnosis of PCLs.

Earlier studies have shown that imaging modalities perform poorly in identifying malignant cysts in the pancreas [6, 14]. This study shows discouraging results as well, with an accuracy of approximately 60% in diagnosing PCLs with high-grade dysplasia or adenocarcinoma for both cross-sectional imaging and EUS-FNA-CEA. Some of the reasons for this result are the moderate frequency of malignancy in small morphologically benign-appearing cysts and the focal presence of high-grade dysplasia in PCLs, which may be missed with fine-needle aspiration. This highlights the need for other markers, such as proteomic markers, to detect PCLs with high-grade dysplasia and pancreatic adenocarcinoma [32]. Additional research is required to improve the detection of these lesions.

The strength of this study is that it is a prospective, long-term study of 10 years. It was conducted at a tertiary referral centre serving a population of two million inhabitants. Experienced specialists in radiology, endoscopy, cytology and pathology were involved. Furthermore, the full potential of EUS was utilized with EUS morphology, cytology and CEA. The gold standard, surgical histology, is robust.

One of the limitations of the study is that it is a single-centre study. The study consists exclusively of patients who underwent surgical resection, reflecting the results for this group of patients and not all patients with PCLs. Although earlier studies have shown that the accuracy rates of CT and MRI are similar in the characterization of PCLs, the fact that our patients underwent either CT or MRI scans may have introduced heterogeneity in the population. Conclusive results for cross-sectional imaging, cytology and CEA were available for the majority but not all patients. However, this result reflects the conditions in real clinical practice.

In conclusion, this study assessed the diagnostic accuracy of EUS-FNA, including cyst fluid analysis, and cross-sectional imaging in cystic lesions of the pancreas. The results show that EUS-FNA with cyst fluid analysis for cytology and CEA is accurate, and it has a higher diagnostic yield than does cross-sectional imaging. Therefore, EUS-FNA with cyst fluid analysis should be considered a complementary test to improve the diagnosis of PCLs. However, the detection of PCLs with high-grade dysplasia or adenocarcinomas needs to be improved. 

## Electronic supplementary material

Below is the link to the electronic supplementary material.

Supplementary Material 1 (5406 kb)
