# Supplementary material for: EUS is accurate in characterizing pancreatic cystic lesions; a prospective comparison with cross-sectional imaging in resected cases
Source: Surg Endosc. 2020 Dec 1;35(12):6650–9. doi: 10.1007/s00464-020-08166-3 (PMC8599246; doi:10.1007/s00464-020-08166-3)
Supplement: Supplementary file 1 — Supplementary Material 1 (5406 kb) [file 464_2020_8166_MOESM1_ESM.docx]

| Patient  no. | Diagnosis according to | |  |  |  |  |
| --- | --- | --- | --- | --- | --- | --- |
|  | **Surgical**  **Pathology** | **CT/MRI** | **EUS-FNA-CEA** | **EUS**  **Morphology** | **EUS**  **Cytology** | **EUS**  **CEA (ng/ml)** |
| 1 | IPMN, L/IGD | Inconclusive | IPMN | IPMN | Mucinous, L/IGD | No CEA |
| 2 | IPMN, L/IGD | Inconclusive | Adenocarcinoma | IPMN/MCN | Nonmucinous, No atypia | 6022 |
| 3 | IPMN, L/IGD | Inconclusive | IPMN | IPMN | Mucinous, No atypia | No CEA |
| 4 | IPMN, L/IGD | Inconclusive | IPMN | IPMN | Mucinous, No atypia | 21 |
| 5 | IPMN, L/IGD | Inconclusive | IPMN | IPMN | Nonmucinous, L/IGD | No CEA |
| 6 | IPMN, L/IGD | Inconclusive | Adenocarcinoma | IPMN | Mucinous, L/IGD | 1642 |
| 7 | IPMN, L/IGD | IPMN | IPMN | IPMN | Mucinous, L/IGD | No CEA |
| 8 | IPMN, L/IGD | IPMN | IPMN | IPMN | Mucinous, No atypia. | 400 |
| 9 | IPMN, L/IGD | IPMN | IPMN | IPMN | Mucinous, No atypia. | No CEA |
| 10 | IPMN, L/IGD | IPMN | Adenocarcinoma | IPMN | Mucinous, No atypia. | 2793 |
| 11 | IPMN, L/IGD | IPMN | Adenocarcinoma | Adenocarcinoma | No puncture | No CEA |
| 12 | IPMN, L/IGD | IPMN | IPMN | IPMN | Mucinous, No atypia. | No CEA |
| 13 | IPMN, L/IGD | IPMN | IPMN | IPMN | Mucinous, No atypia | 210 |
| 14 | IPMN, L/IGD | IPMN | Adenocarcinoma | IPMN | Mucinous, No atypia | 1060 |
| 15 | IPMN, L/IGD | IPMN | IPMN | IPMN | Nonmucinous, No atypia | No CEA |
| 16 | IPMN, L/IGD | IPMN | IPMN | IPMN | Mucinous, No atypia | 17 |
| 17 | IPMN, L/IGD | IPMN | IPMN | IPMN | Nonmucinous, No atypia | 44 |
| 18 | IPMN, L/IGD | IPMN | IPMN | IPMN | Mucinous, No atypia | 370 |
| 19 | IPMN, L/IGD | IPMN | IPMN | IPMN | Unrepresentative | 730 |
| 20 | IPMN, L/IGD | IPMN | IPMN | IPMN | Unrepresentative | 12 |
| 21 | MCN, L/IGD | IPMN | IPMN | IPMN | Mucinous, No atypia | 30 |
| 22 | IPMN, L/IGD | IPMN | IPMN | IPMN | Mucinous, L/IGD | 42 |
| 23 | IPMN, L/IGD | IPMN | Adenocarcinoma | IPMN | Mucinous, L/IGD | 2322 |
| 24 | IPMN, L/IGD | IPMN | IPMN | IPMN | Mucinous, No atypia | 7 |
| 25 | IPMN, HGD | Inconclusive | IPMN | SCN | Mucinous, L/IGD | 25 |
| 26 | IPMN, HGD | MCN | IPMN | IPMN | Mucinous, No atypia | No CEA |
| 27 | IPMN, HGD | IPMN | IPMN | IPMN | Mucinous, No atypia | 83 |
| 28 | IPMN, HGD | IPMN | IPMN | IPMN | Mucinous, No atypia | No CEA |
| 29 | IPMN, HGD | IPMN | IPMN | IPMN | Mucinous, No atypia | No CEA |
| 30 | IPMN, HGD | IPMN | IPMN | IPMN | Nonmucinous, No atypia | No CEA |
| 31 | IPMN, HGD | IPMN | IPMN | IPMN | Mucinous, No atypia | No CEA |
| 32 | IPMN, HGD | IPMN | IPMN | IPMN | Mucinous, No atypia | No CEA |
| 33 | IPMN, HGD | IPMN | IPMN | IPMN | Mucinous, L/IGD | No CEA |
| 34 | IPMN, HGD | IPMN | IPMN | IPMN | Mucinous, L/IGD | 121 |
| 35 | Adenocarcinoma | IPMN | Adenocarcinoma | Adenocarcinoma | No puncture | No CEA |
| 36 | Adenocarcinoma | IPMN | Adenocarcinoma | Adenocarcinoma | Adenocarcinoma | 6990 |
| 37 | Adenocarcinoma | IPMN | Adenocarcinoma | IPMN | Adenocarcinoma | 232 |
| 38 | Adenocarcinoma | IPMN | IPMN | IPMN | Unrepresentative | 129 |
| 39 | Adenocarcinoma | IPMN | IPMN | IPMN | Mucinous, No atypia | 634 |
| 40 | Adenocarcinoma | IPMN | IPMN | IPMN | Nonmucinous, No atypia | No CEA |
| 41 | Adenocarcinoma | Adenocarcinoma | Adenocarcinoma | Unknown | Adenocarcinoma | No CEA |
| 42 | Adenocarcinoma | IPMN | Adenocarcinoma | Adenocarcinoma | Mucinous, L/IGD | 162 |
| 43 | Adenocarcinoma | IPMN | IPMN | IPMN | Unrepresentative | 20 |
| 44 | Adenocarcinoma | IPMN | Adenocarcinoma | Adenocarcinoma | Adenocarcinoma | 420 |
| 45 | Adenocarcinoma | IPMN | Adenocarcinoma | Adenocarcinoma | Mucinous, HGD | <0.5 |
| 46 | SCN | Inconclusive | SCN | SCN | Unrepresentative | 10 |
| 47 | SCN | Inconclusive | SCN | IPMN | Nonmucinous, No atypia | <0.5 |
| 48 | SCN | Inconclusive | SCN | IPMN | Nonmucinous, No atypia | <0.5 |
| 49 | SCN | IPMN | SCN | IPMN | Nonmucinous, No atypia | <0.5 |
| 50 | SCN | IPMN | SCN | IPMN | Unrepresentative | <0.5 |
| 51 | Pseudocyst | Pseudocyst | Pseudocyst | Pseudocyst | Pseudocyst | 66 |
| 52 | NET | NET | NET | NET | NET | No CEA |
| 53 | NET | MCN | NET | NET | NET | 80 |
| 54 | SPN | Inconclusive | SCN | Unknown | Nonmucinous, No atypia | <0.5 |
| 55 | SPN | Inconclusive | IPMN | IPMN | Mucinous, No atypia | 125 |
| 56 | Cystic GIST | Cystic GIST | Pseudocyst | Pseudocyst | Nonmucinous, No atypia | 8 |
| 57 | “Cystic ducts” | NET | Adenocarcinoma | IPMN | Adenocarcinoma | 7 |
| 58 | “Cystic ducts” | MCN | Adenocarcinoma | Adenocarcinoma | Nonmucinous/no atypia | 11 |

**Appendix Table 1** Overview of all patients included in the study (*n =* 58).

CT = computed tomography; MRI = magnetic resonance imaging; EUS-FNA-CEA = endoscopic ultrasound morphology, cytology and carcinoembryonic antigen; IPMN = main/branch duct intraductal papillary mucinous neoplasm; MCN = mucinous cystic neoplasm; SCN = serous cystic neoplasm; NET = neuroendocrine tumor; SPN = solid pseudopapillary neoplasm; GIST = gastrointestinal stromal tumor; Cystic ducts = seen in chronic pancreatitis cases; No atypia = no cells or no atypia seen; L/IGD = low- or intermediate-grade dysplasia; HGD =high-grade dysplasia; Unrepresentative = unrepresentative material

**Appendix Fig. 1** Boxplots (median as a line, 50% within the box, whiskers represent 1.5 interquartile range, outliers identiﬁed with circle and star) showing cyst CEA concentrations in mucinous vs non-mucinous PCLs.

CEA = Carcinoembryonic Antigen

Mucinous; 25 percentile: 30, 50 percentile: 187, 75 percentile: 730

Non-mucinous; 25 percentile: .00, 50 percentile: .85, 75 percentile: 38.5

Mucinous = Main/branch duct intraductal papillary mucinous neoplasm (M/BD-IPMN), mucinous cystic neoplasm (MCN) or adenocarcinoma


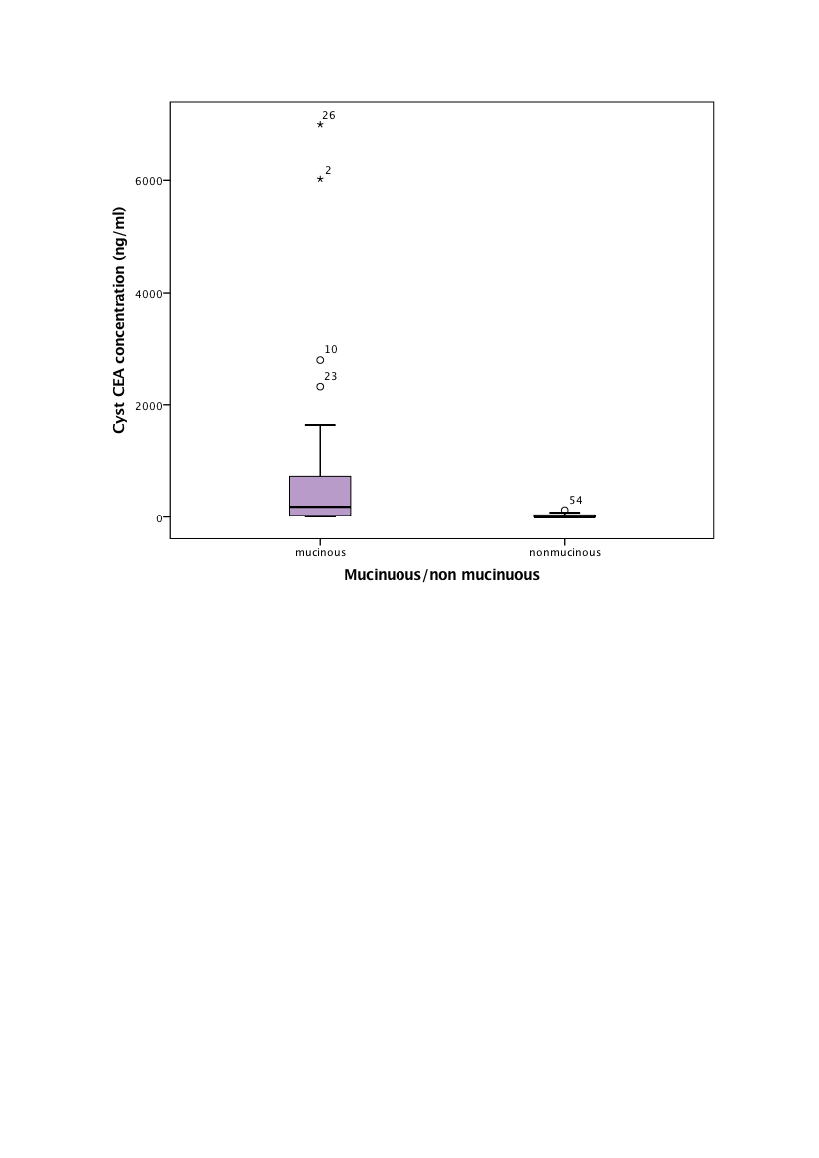


**Appendix Fig. 2** Receiver operating characteristic (ROC) curve to determine the optimal cut-off value for cyst CEA for differentiating between mucinous and non-mucinous PCLs.

AUC, area under the curve = 0.899

Mucinous = Main/branch duct intraductal papillary mucinous neoplasm (M/BD-IPMN), mucinous cystic neoplasm (MCN) or adenocarcinoma


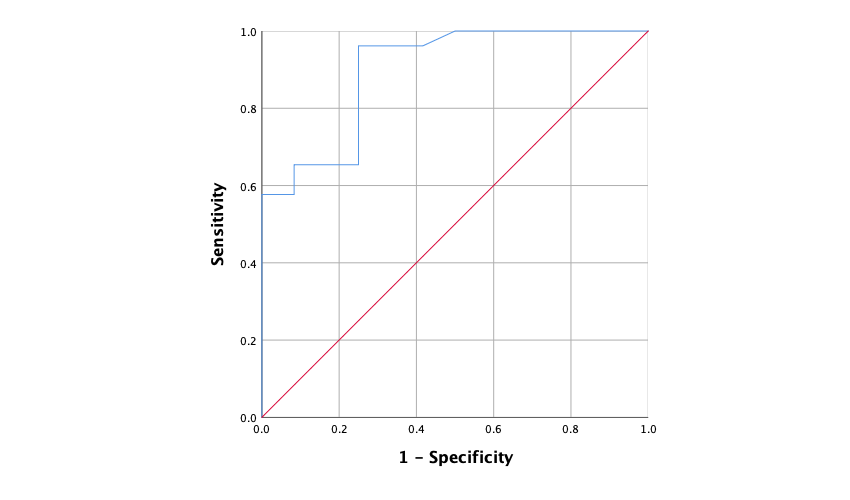


| Patient  no. | Diagnosis according to  Surgical Pathology | EUS Amylase (ukat/L) |
| --- | --- | --- |
| 4 | IPMN, L/IGD | 210 |
| 10 | IPMN, L/IGD | 13.5 |
| 14 | IPMN, L/IGD | 4.6 |
| 16 | IPMN, L/IGD | 317 |
| 17 | IPMN, L/IGD | 7420 |
| 18 | IPMN, L/IGD | <0.1 |
| 19 | IPMN, L/IGD | 7.6 |
| 20 | IPMN, L/IGD | 2450 |
| 25 | IPMN, HGD | 2780 |
| 37 | Adenocarcinoma | 0.9 |
| 38 | Adenocarcinoma | 81 |
| 39 | Adenocarcinoma | 758 |
| 33 | Adenocarcinoma | 3560 |
| 46 | SCN | 0.3 |
| 47 | SCN | 0.8 |
| 48 | SCN | 5.6 |
| 49 | SCN | 0.9 |
| 52 | NET | 1.2 |
| 54 | SPN | 3 |
| 55 | SPN | 236 |
| 56 | Cystic GIST | 0.8 |
| 58 | “Cystic ducts” | 5400 |

**Appendix Table 2** Cyst fluid amylase levels in patients with mucinous and non-mucinous PCLs.

EUS = endoscopic ultrasound; IPMN = main/branch duct intraductal papillary mucinous neoplasm; L/IGD = low- or intermediate-grade dysplasia; HGD =high-grade dysplasia; SCN = serous cystic neoplasm; NET = neuroendocrine tumor; SPN = solid pseudopapillary neoplasm; GIST = gastrointestinal stromal tumor; Cystic ducts = seen in chronic pancreatitis cases;
